# Supplementary material for: Potential Resistance to Oxaliplatin-Based Regimens in Gastric Cancer Patients with ERBB2 R678Q Mutation: Evidence from a National Genomic Database
Source: Curr Issues Mol Biol. 2025 Jun 6;47(6):430. doi: 10.3390/cimb47060430 (PMC12192524; doi:10.3390/cimb47060430)
Supplement: Supplementary file 1 [file cimb-47-00430-s001.zip › cimb-3652778-supplementary.pdf]

Supplementary Table S1. Patients background of registered in the Center for Cancer Genomics and Advanced Therapeutics database.

Total Cases (n = 92,802)			
**Primary Site**		**Cancer Genomics Test**	
Colorectal	15155	FoundationOne CDx	64477
Pancreas	14376	FoundationOne Liquid CDx	14156
Bile Duct	8134	NCC Oncopanel System	8901
Breast	6955	GenMine™ TOP Cancer Panel	3478
Prostate	5647	Guardant360 CDx	1790
Esophagus/Stomach	5614		
Lung	5541	**Age Group (years)**	
Ovary/Fallopian Tube	5039	70-79	27146
Soft Tissue	3590	60-69	26074
Uterus	3119	50-59	19491
Others	19632	40-49	9767
		80-89	4265
**Sex**		30-29	3201
Male	46461	20-29	1071
Female	46336	10-19	930
Unknown	5	0-9	791
		90-	66

The study periods for each genomic testing were as follows: NCC Oncopanel System (June 1, 2019 to February 14, 2025), FoundationOne® CDx (June 1, 2019 to February 15, 2025), FoundationOne® Liquid CDx (August 1, 2021 to February 15, 2025), Guardant360® CDx (July 24, 2023 to February 14, 2025), and GenMineTM TOP Cancer Panel (August 1, 2023 to February 14, 2025).

Supplementary Table S2. Esophagus/Stomach cases background of registered in the Center for Cancer Genomics and Advanced Therapeutic data.

Esophagus/Stomach Cases (n=5,614)			
**Cancer Genomics Test**		**Age Group (years)**	
FoundationOne CDx	4139	70-79	1809
FoundationOne Liquid CDx	626	60-69	1788
NCC Oncopanel System	619	50-59	1066
GenMine™ TOP Cancer Panel	144	40-49	490
Guardant360 CDx	86	80-89	215
		30-29	185
**Sex**		20-29	52
Male	4040	10-19	7
Female	1574	0-9	1
		90-	1

The study periods for each genomic testing were as follows: NCC Oncopanel System (June 1, 2019 to February 14, 2025), FoundationOne®

CDx (June 1, 2019 to February 15, 2025), FoundationOne® Liquid CDx (August 1, 2021 to February 15, 2025), Guardant360® CDx (July 24, 2023 to February 14, 2025), and GenMineTM TOP Cancer Panel (August 1, 2023 to February 14, 2025).

Supplementary Table S3. Gastric or gastroesophageal junction adenocarcinomas with *ERBB2* amplification background of registered in the Center for Cancer Genomics and Advanced Therapeutic Database.

**Gastric or Gastroesophageal Junction Adenocarcinoma *ERBB2* amplification Cases (N=503)**			
**Cancer Genomics Test**		**Treatment Response to Oxaliplatin***	
FoundationOne CDx	430	Complete Response	7
FoundationOne Liquid CDx	36	Partial Response	163
NCC Oncopanel System	25	Stable Disease	109
GenMine™ TOP Cancer Panel	10	Progressive Disease	75
Guardant360 CDx	2	Not Evaluated	64
**Sex**		**Treatment Response to Ramucirumab***	
Male	384	Complete Response	2
Female	119	Partial Response	85
		Stable Disease	19
**Age Group (years)**		Progressive Disease	553
70-79	200	Not Evaluated	97
60-69	147		
50-59	81	**Treatment Response to Nivolumab***	
40-49	27	Complete Response	3
80-89	23	Partial Response	52
30-39	19	Stable Disease	88
20-29	5	Progressive Disease	114
10-19	1	Not Evaluated	59

*: Regimen containing indicated drug. The study periods for each genomic testing were as follows: NCC Oncopanel System (June 1, 2019 to February 14, 2025), FoundationOne® CDx (June 1, 2019 to February 15, 2025), FoundationOne® Liquid CDx (August 1, 2021 to February 15, 2025), Guardant360® CDx (July 24, 2023 to February 14, 2025), and GenMineTM TOP Cancer Panel (August 1, 2023 to February 14, 2025).
